# IPRS: Leveraging Gene-Environment Interaction to Reconstruct Polygenic Risk Score

**DOI:** 10.3389/fgene.2022.801397

**Published:** 2022-03-24

**Authors:** Yingdan Tang, Dongfang You, Honggang Yi, Sheng Yang, Yang Zhao

**Affiliations:** ^1^ Department of Biostatistics, School of Public Health, Nanjing Medical University, Nanjing, China; ^2^ Center of Biomedical Big Data and the Laboratory of Biomedical Big Data, Nanjing Medical University, Nanjing, China; ^3^ Jiangsu Key Lab of Cancer Biomarkers, Prevention and Treatment, Collaborative Innovation Center for Cancer Personalized Medicine, Nanjing Medical University, Nanjing, China

**Keywords:** polygenic risk score, gene-environment interaction, genome-wide association analysis, prediction model, risk stratification

## Abstract

**Background:** Polygenic risk score (PRS) is widely regarded as a predictor of genetic susceptibility to disease, applied to individuals to predict the risk of disease occurrence. When the gene-environment (G×E) interaction is considered, the traditional PRS prediction model directly uses PRS to interact with the environment without considering the interactions between each variant and environment, which may lead to prediction performance and risk stratification of complex diseases are not promising.

**Methods:** We developed a method called interaction PRS (iPRS), reconstructing PRS by leveraging G×E interactions. Two extensive simulations evaluated prediction performance, risk stratification, and calibration performance of the iPRS prediction model, and compared it with the traditional PRS prediction model. Real data analysis was performed using existing data from the Prostate, Lung, Colorectal, and Ovarian (PLCO) Cancer Screening Trial study to predict genetic susceptibility, pack-years of smoking history, and G×E interactions in patients with lung cancer.

**Results:** Two extensive simulations indicated iPRS prediction model could improve the prediction performance of disease risk, the accuracy of risk stratification, and clinical calibration performance compared with the traditional PRS prediction model, especially when antagonism accounted for the majority of the interaction. PLCO real data analysis also suggested that the iPRS prediction model was superior to the PRS prediction model in predictive effect (*p* = 0.0205).

**Conclusion:** IPRS prediction model could have a good application prospect in predicting disease risk, optimizing the screening of high-risk populations, and improving the clinical benefits of preventive interventions among populations.

## Introduction

With the rapid development of the genome in recent years, genome-wide association studies (GWAS) have shown that complex diseases are polygenic. (Yang et al., 2010; Visscher et al., 2017; Sullivan and Geschwind, 2019) As more and more disease susceptibility sites were discovered, more than 15,000 genetic susceptibility loci associated with the disease have been identified (Buniello et al., 2019), it is possible to use genetic data to predict and to stratify disease risk (Khera et al., 2018; Wray et al., 2018; Mavaddat et al., 2019), as well as to screen high-risk individuals group of diseases. (Lewis and Vassos, 2020) To synthesize information from multiple loci, Purcell et al. first proposed polygenic score (PGS) for schizophrenia. (Consortium et al., 2009) It also refers to polygenic risk score (PRS), when the interesting trait is binary. (Fritsche et al., 2019; Owens et al., 2019) PRS aims to quantify the cumulative effect of multiple genomic variants into a score to predict the genetic susceptibility of disease. The construction of a typical PRS usually has two steps: the first is the process of “variants selection” to determine the sites to be included in the model; The second is “weight estimation”, the process of obtaining the coefficients or weights attached to the selected variants. (Hang and Shen, 2019) Moreover, since the emergence of efficient software [i.e., GCTA (Yang et al., 2011) and GEMMA (Zhou and Stephens, 2012)], the best linear unbiased predictor (BLUP) was widely used to define significant SNP and construct PRS in the human genome. Accurate construction of PRS can facilitate disease prevention and intervention at an early stage and can aid in the development of personalized medicine. (Yang and Zhou, 2020)

Interactions between genes and environmental factors also contributed to the generation and development of complex diseases (Torkamani et al., 2018; Wang et al., 2019), and could explain some proportion of individual differences of complex diseases. Many studies have reported that environmental factors modified the effect of PRS. As an example, the study of Shi et al. showed that hormonal birth control in women decreased the risk of PRS for Young-onset breast cancer (YOBC). (Shi et al., 2020)

When considering the interaction between genetic risk score and environment, most studies directly use traditional PRS to interact with the environment. (Li et al., 2015; Peyrot et al., 2018; Guloksuz et al., 2019) However, during the construction procedure of traditional PRS, variants and their corresponding weights are determined without considering the G×E interactions. Thus, some SNPs potentially interacted with environmental factors maybe fail to be identified if they do not have main effects, or their direction of interaction effect is different from that of the main effect. Meanwhile, the weights of identified SNPs are the accumulations of main effects and interaction effects, leading to inaccurate estimation and, risk predictions. Furthermore, some SNPs in the PRS may interact with the environment in antagonistic, while some others in synergistic ways. Therefore, the direct use of PRS to interact with the environment may make antagonistic interaction and synergistic interaction cancel each other out.

Here, we propose to construct PRS that consider both the main effect of the SNP and their interactions with environmental factors, referring to interaction PRS (iPRS). Simulations were performed under different model assumptions to evaluate the performance of the iPRS prediction model. We also performed real data application in the Prostate, Lung, Colorectal, and Ovarian (PLCO) Cancer Screening Trial study. We provided implemented iPRS prediction model (https://github.com/predictionmodel/IPRS).

## Materials and Methods

### PRS Prediction Model

The marginal effect is the conditional effect of one variant averaging across the different levels of the other variants. (Williams, 2012) Suppose that the outcome is binary and we are using the following logistic model to identify the association between genes and disease,

$$\text{logit}P\left(y_{i}=1\right)=\alpha_{ij}+\beta_{G_{ij}}^{M}G_{ij}+\beta_{E_{ij}}E_{i}+\beta_{C_{ij}}C_{i}$$ (1)

Where $y_{i}$ is binary disease outcome of the $i^{th}$ sample; $G_{ij}$ is the number of risk alleles (coded as 0, 1, 2) of the $j^{th}$ variant; $\beta_{G_{ij}}^{M}$ , marginal effect, is the effect of the $j^{th}$ SNP conditional to $E_{i}$ ; $\beta_{E_{ij}}$ is the environment variable’s effect; $\beta_{C_{ij}}$ is the effect of other covariates $C_{i}$ ; $\alpha_{ij}$ is an intercept.

PRS for an individual is the summation of risk variants that have been identified in GWAS. In its simplest and most common form, PRS is defined as the weighted sums of marginal effects of SNPs. PRS is shown as follows:

$$PRS_{i}=\sum_{j=1}^{J}\beta_{G_{\text{ij}}}^{M}G_{ij}$$ (2)

Where $\beta_{G_{ij}}^{M}$ is the estimated effect size of the $j^{th}$ SNP (usually obtained from summary statistics) and $G_{ij}$ indicates the genotype of $j^{th}$ SNP for an individual. In this way, PRS aggregate the contribution of an individual’s germline genome into a single number proportional to the risk for a given disease. (Lambert et al., 2019)

When considering the interaction between PRS and environmental factors, most studies construct the prediction model as follows:

$$\text{logit}P\left(y_{i}=1\right)=PRS_{i}+PRS_{i}\times E_{i}+E_{i}$$ (3)

As we have stated above, using PRS directly to interact with the environment and build predictive models may lead to potentially biased results.

### IPRS Prediction Model

The G×E interaction model included SNP, environmental factor, and their interaction term in the model. Especially, when interactions are included in the model, the average conditional effect of this variant at different levels of other variants is called the main effect. (Pandis et al., 2014) The traditional interaction model is shown as follows:

$$\text{logit}P\left(y_{i}=1\right)=\alpha_{ij}+\beta_{G_{ij}}^{M}G_{ij}+\beta_{E_{ij}}E_{i}+\beta_{G_{ij}\times E}^{I}G_{ij}\times E_{i}+\beta_{C_{ij}}C_{i}$$ (4)

Where $\beta_{G_{ij}}^{M}$ is the main effect of the $j^{th}$ SNP; $\beta_{G_{ij}\times E}^{I}$ is the interaction between the $j^{th}$ SNP and environmental variant $E_{i}$ .

The iPRS is constructed using both SNPs and SNP×E interactions. The weight of each SNP is the main effect of each SNP after considering SNP×E interaction in the regression, and the weight of each SNP×E interaction term is the interaction between each SNP and the environment as:

$$IPRS_{i}=\sum_{j=1}^{J}\beta_{G_{ij}}^{M}G_{ij}+\sum_{j=1}^{J}\beta_{G_{ij}\times E}^{I}G_{ij}\times E_{i}$$ (5)

Where $\beta_{G_{ij}}^{M}$ is the main effect of the $j^{th}$ SNP of the $i^{th}$ sample; $\beta_{G_{ij}\times E}^{I}$ is the interaction between the $j^{th}$ SNP and environmental variant $E_{i}$ ; $\beta_{G_{ij}\times E}^{I}G_{ij}\times E_{i}$ explains the effect of the $j^{th}$ SNP at different levels of environmental variant. All alleles are all flipped into risk alleles.

To predict the disease risk, an iPRS predictive model can be written as follows:

$$\text{logit}P\left(y_{i}=1\right)=IPRS_{i}+E_{i}$$ (6)

### Simulations

We designed two simulations to compare the predictive performances of the iPRS prediction model to that of the PRS prediction model in the presence of G×E interactions.

Following the sparse assumption [i.e. clumping and threshold (CT) and stacked clumping and thresholding (SCT), the most commonly used method for computing PRS (Privé et al., 2019)], simulation I assumed that a small number of SNP contributed to a specific disease and that the effects of them are treated as a fixed effect. (Privé et al., 2019) First, we simulated 10 SNPs from a Beta distribution of B (2, MAF), and MAF was generated from a uniform distribution of U (0.1, 0.5). We hypothesized that these SNPs were obtained by genome-wide association studies (GWAS) that statistically associated with some diseases (that is, *p* value <5 × 10^−8^), some of which may be causal SNPs that actually influence disease, and some of which may be false positives. The environmental variant was generated from B (1, 0.5). The binary phenotype was generated as follows:

$$\text{logit}P\left(y_{i}=1\right)=\sum_{j=1}^{10}\beta_{i}^{M}{\mathrm{SNP}}_{i}+\sum_{j=1}^{10}\beta_{i}^{I}{\mathrm{SNP}}_{i}\times E_{i}+\beta_{E_{ij}}E_{i}$$ (7)

Where $y_{i}$ is the simulated phenotype (i.e., diseases onset or not) of the $i^{th}$ sample; ${\mathrm{SNP}}_{i}=\left(SNP_{i1},SNP_{i2},\cdots ,SNP_{i10}\right)$ ; $E_{i}$ is the environmental risk factor. $\beta_{i}^{M}$ are the main effects of ${\mathrm{SNP}}_{i}$ . We randomly set six causal risk SNPs whose main effects were not 0, and the main effects of the remaining SNPs were all 0; $\beta_{i}^{I}$ are the interactions of SNP×E interaction terms. We also randomly set four SNPs from the SNPs with the main effect to have non-zero interactions with environment variant, the rest of the interactions were all 0.

We simulated three different scenarios: (I) interactions between SNPs and the environmental factor are all antagonistic; (II) interactions between SNPs and the environmental factor are all synergistic; (III) half of interactions are antagonistic, and half are synergistic. For each simulation setting, we generated 1,000 simulated datasets. Each simulated dataset includes 1,000 cases and 1,000 controls. Details of simulation I are shown in Supplementary Table S1.

Following the polygenic assumption [i.e., BLUP, through fitting a genomic relationship matrix to estimate genetic effect to construct PRS (De Los Campos et al., 2013; Truong et al., 2020)], simulation II assumed that all variants contribute to the disease with a small effect size. To make the simulation closer to a real scenario, we used all SNPs on chromosome 1 from PLCO Cancer Screening Trial and simulated environmental variable and phenotype with 14,415 individuals to generate simulation datasets. After QC, a total of 14,415 individuals and 438,548 SNPs were included in the simulation. We simulated the causal effects of SNPs from a normal distribution $N\left(0,h^{2}/m\right)$ , where m is the number of SNPs and $\left(h^{2}/m\right)$ is the variance. We set $h^{2}$ to be 0.3 representing moderate heritability and set m to 1% of all 438,548 SNPs. Meanwhile, we randomly selected 0.1‰ of all SNPs to have interactions, antagonistic interactions accounted for 70% of all interactions. The simulation of the environmental factor was the same as that in simulation I. We repeated the simulation 100 times.

### Real Data Analysis With PLCO

We also evaluated the iPRS prediction model by real data within the PLCO Cancer Screening Trial study. The PLCO Cancer Screening Trial study was a large randomized trial designed and sponsored by the National Cancer Institute (NCI) to determine the effects of screening on cancer-related mortality and secondary endpoints in men and women aged 55–74. (Gohagan et al., 2000) Previous studies have reported that smoking is a risk factor for lung cancer, and some studies have shown that there are interactions between pack-years of smoking and susceptibility SNPs of lung cancer, and interactions between some SNPs and lung cancer are antagonistic. (Zhang et al., 2014) Thus, in this real data analysis, we constructed and evaluated the iPRS prediction model and traditional PRS prediction model for lung cancer participants with susceptibility SNPs of lung cancer and pack-years of smoking. The process was described below.

For SNP quality control (QC), we focused on our analysis on autosome SNPs following the standard QC procedure used in Landi et al’s study. (Landi et al., 2009) Samples were screened and selected only if they had a minimum 95% successful genotype call rate. We filtered out SNPs *1*) with a minor allele frequency (MAF) < 0.01, *2*) with a Hardy-Weinberg (HWE) test *p* value <10^−6^, *3*) with a proportion of missingness (Pm) > 0.05, or *4*) that are a duplicated SNP or related SNP. After these QC steps, we retained a total of 14,415 individuals and 5,685,769 SNPs for analysis.

We obtained genotype, pack-years of smoking history, and confirmed lung cancer from PLCO with 14,415 individuals. Through Logistic regression considering interaction, we obtained the main effect of all SNPs and the interactions between SNPs and pack-years of smoking history. In order to include more SNPs with interaction, we derived the SNPs for the construction of iPRS prediction model specific for PLCO population from SNPs that *p* value of main effect or interaction less than 5 × 10^−6^ in PLCO study. After selection, 299 risk variants were kept for the calculation of the iPRS prediction model. Janjigian et al’s study showed that lung cancer patients with ≤15 pack-years histories of smoking had longer median survival than patients who had smoked >15 pack years. (Janjigian et al., 2010) So, pack-years of smoking history translated into binary variant depending on whether they were longer than 15 years. The 14,415 participants included 1,453 cases and 12,962 controls.

We used 5-fold cross-validation to train and evaluate the iPRS prediction model and traditional PRS prediction model, and evaluated two prediction models described above in terms of their prediction accuracy, risk stratification, and calibration performance. We used the area under the receiver operation curve (AUC) to compare the prediction performance of the iPRS prediction model and PRS prediction model and compared the difference between them with the Delong algorithm (Sun and Xu, 2014). The risk stratification was reflected in the prevalence rates of different risk populations, and the chi-square test was used to compare the prevalence of the iPRS prediction model and PRS prediction model. We used the Brier score and calibration curve to measure the calibration performance of the iPRS prediction model and PRS prediction model. (Huang et al., 2020)

All analyses and figures were performed using R, version 3.2.0 (R Foundation for Statistical Computing, Vienna, Austria). Power calculations were performed using G*Power, version 3.1 (Faul, Erdfelder, Lang, and Buchner) (Faul et al., 2009).

## Results

### Simulations

**FIGURE 1:**
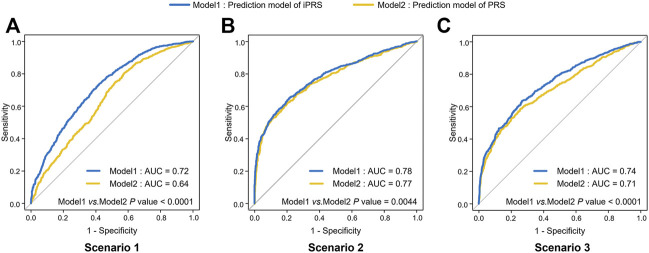
ROC of iPRS prediction model and PRS prediction model in three scenarios of simulations I with independent SNPs. **(A)** Scenario 1: SNPs are all risk factors, the environmental variant is a risk factor, interactions are antagonistic; **(B)** Scenario 2: SNPs are all risk factors, the environmental variant is a risk factor, interactions are synergistic; **(C)** Scenario 3: SNPs are all risk factors, the environmental factor is a risk variant, half of the interactions are antagonistic, and half are synergistic. The *p* values were shown that the results of comparing the AUC of iPRS prediction model and traditional PRS prediction model.

In simulation I, the AUC of iPRS prediction model average improves 6.0% than that of the traditional model in the three scenarios (Figure 1). As expectation, we found that the average AUC of iPRS prediction model was 0.72 in scenario 1 (antagonistic interactions), 0.78 in scenario 2 (synergistic interactions), 0.74 in scenario 3 (half antagonistic interactions and half synergistic interactions), which were all significantly higher than that of PRS prediction model (*p* < 0.0001, *p* = 0.0044, *p* < 0.0001 respectively).

**FIGURE 2:**
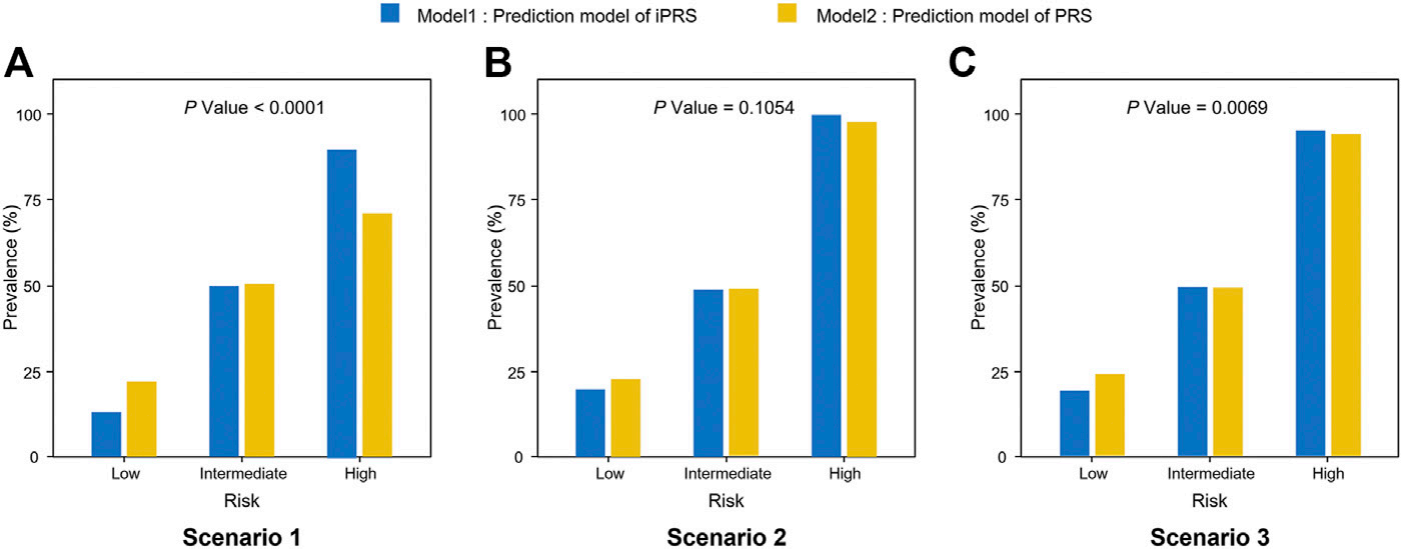
Prevalence of high risk, intermediate risk, low risk population according to the predictive value of iPRS prediction model or PRS prediction model in three scenarios of simulations I with independent sparse SNPs. Samples were defined as high risk, intermediate risk, and low risk populations according to the top 5%, 5%–95%, and the bottom 5% of the predictive value of iPRS prediction model or PRS prediction model. Significant differences in risk categories of the population were noted. **(A)** Scenario 1: SNPs are all risk factors, the environmental variant is a risk factor, interactions are antagonistic; **(B)** Scenario 2: SNPs are all risk factors, the environmental variant is a risk factor, interactions are synergistic; **(C)** Scenario 3: SNPs are all risk factors, the environmental factor is a risk variant, half of the interactions are antagonistic, and half are synergistic.

Furthermore, iPRS prediction model improves the accuracy of risk stratification and is more helpful to screening for high-risk individuals. According to the study of Dai et al., the top 5%, medium 90%, and bottom 5% of the predictive value of iPRS prediction model or PRS prediction model were used to define high, intermediate, low genetic risk populations, respectively. (Dai et al., 2019) The accuracy of risk stratification of iPRS prediction model was better than that of PRS prediction model. The result showed that the accuracy of risk stratification of iPRS prediction model was better than that of PRS prediction model in scenarios 1 and scenarios 3 (*p* < 0.0001, *p* = 0.0069 respectively) (Figure 2). When there was only synergism (scenario 2), the improvement of risk stratification of iPRS prediction model was not significant (*p* = 0.1054).

**FIGURE 3:**
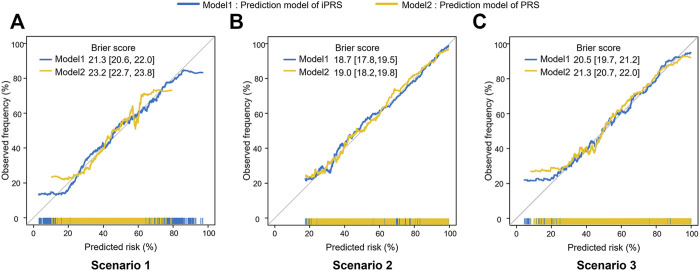
Calibration Plots and Brier scores of iPRS prediction model and PRS prediction model in three scenarios of simulations I with independent sparse SNPs. Calibration plots summarize the graphical agreement between observed and predicted risks. In an ideal model, pairs of the observed and predicted risks lie on a 45-degree angle line. Curves falling under the 45-degree angle line indicate that predicted risks overestimate (are higher than) observed risks, while curves falling above the 45-degree angle line indicate that predicted risks underestimate (are lower than) observed risks. **(A)** Scenario 1: SNPs are all risk factors, the environmental variant is a risk factor, interactions are antagonistic; **(B)** Scenario 2: SNPs are all risk factors, the environmental variant is a risk factor, interactions are synergistic; **(C)** Scenario 3: SNPs are all risk factors, the environmental factor is a risk variant, half of the interactions are antagonistic, and half are synergistic. The numbers in square brackets represent 95%CI of Brier score.

The iPRS prediction model could improve the calibration performance and make the predicted risk more likely to be true observed frequency. In scenario 1 of simulation I, the Brier score of iPRS prediction model was significantly lower than that of PRS prediction model, indicating iPRS prediction model had a smaller mean square error between the actual outcome and the estimated probabilities than the PRS prediction model (Figure 3). Moreover, the iPRS prediction model could also account for more predicted probabilities. The Brier scores and calibration curves in scenario 2 and scenario 3 were similar to those from scenario 1. Simulation I assumed that SNPs having interactions with the environmental factor all had main effects, the results of additional simulation, which assumed that part of the SNPs interacting with environmental factor had no main effects, were similar to simulation I and presented at Supplementary Figures S2–S5.

**FIGURE 4:**
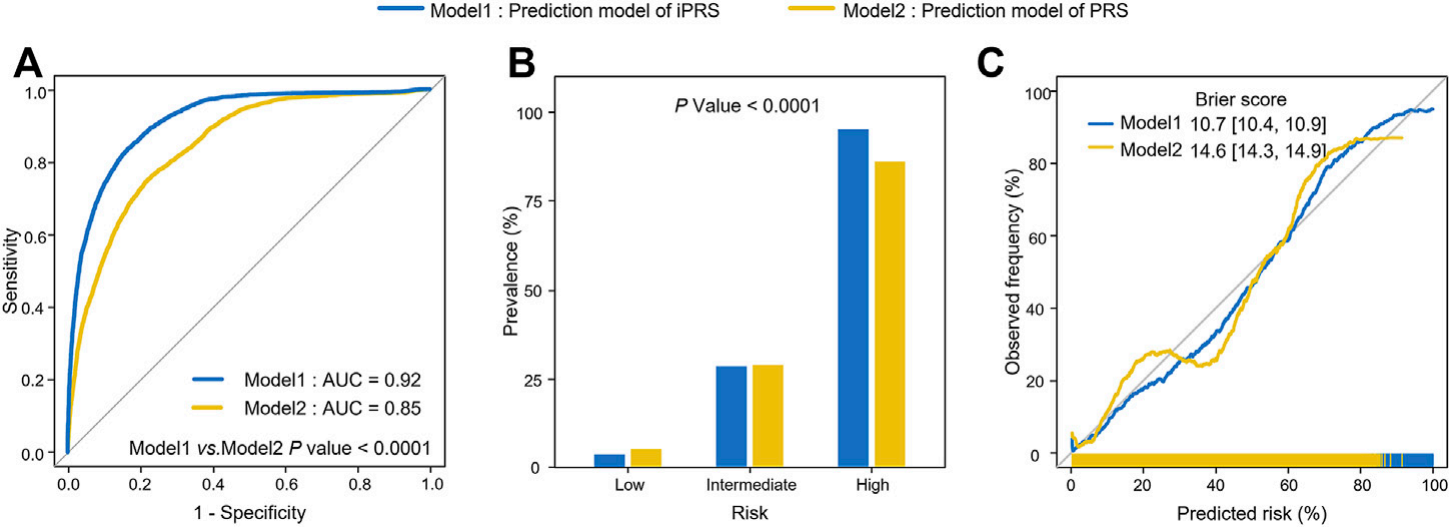
Analysis of iPRS prediction model in prediction performance, risk stratification, calibration performance for simulation II with real genotypes. **(A)** AUCs and ROC curves of iPRS prediction model and PRS prediction model. **(B)** Prevalence of high risk, intermediate risk, low risk population stratified by the predictive value of iPRS prediction model or PRS prediction model in simulations II. Samples were defined as high risk, intermediate risk, and low risk populations according to the top 5%, 5%–95%, and the bottom 5% of the predictive value of iPRS prediction model or PRS prediction model. **(C)** Calibration Plots and Brier scores of iPRS prediction model or PRS prediction model.

In simulation II, the average AUC of iPRS prediction model was 0.92, while the average AUC of PRS prediction model was 0.85, and the difference between them was statistically significant (*p* < 0.0001) (Figure 4A). The accuracy of risk stratification of iPRS prediction model was significantly better than that of PRS prediction model (*p* < 0.0001) (Figure 4B). For calibration result, compared with traditional PRS prediction model, the calibration curve of iPRS prediction model was closer to the diagonal line, indicating that the disease risk of different populations predicted by iPRS prediction model was more consistent with the observed disease frequency. Furthermore, the calibration curve of iPRS prediction model was longer than that of PRS prediction model, indicating that iPRS prediction model explained more predictive risk than PRS prediction model. Accordingly, the Brier score of iPRS prediction model was significantly lower than that of traditional PRS prediction model (Figure 4C).

### PLCO Application

**FIGURE 5:**
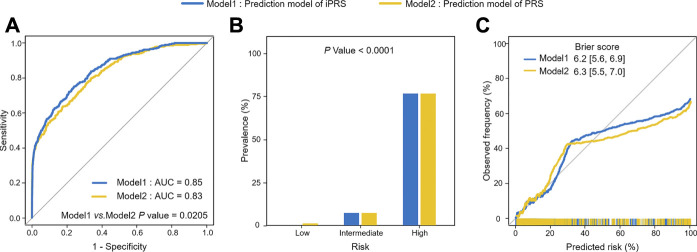
Analysis of iPRS prediction model in prediction performance, risk stratification, calibration performance for lung cancer in the application of PLCO for 5-fold cross-validation. **(A)** AUCs and ROC curves of iPRS prediction model and PRS prediction model applied for PLCO dataset. **(B)** Lung cancer prevalence of high risk, intermediate risk, low risk population stratified by the predictive value of iPRS prediction model or PRS prediction model in the application of PLCO. Samples were defined as high risk, intermediate risk, and low risk populations according to the top 5%, 5%–95%, and the bottom 5% of predictive value of iPRS prediction model or PRS prediction model. **(C)** Calibration Plots and Brier scores of iPRS prediction model and PRS prediction model for lung cancer in the application of PLCO.

For the application, we used 1,453 lung cancer patients and 12,962 individuals without lung cancer and used 299 SNPs associated with lung cancer risk or interacting with pack-years of smoking history in the PLCO study. The results of 5-fold cross-validation showed that iPRS prediction model was significantly superior to traditional PRS prediction model in terms of prediction performance and risk population of lung cancer risk. The AUC of iPRS prediction model was 0.85, significantly greater than that of PRS prediction model (*p* = 0.0205) (Figure 5A). For risk stratification, the prevalence of high-risk population of iPRS prediction model and PRS prediction model was 76.55%, with almost no difference between the two models. However, the prevalence of low-risk population was almost zero, and that of PRS prediction model was 6.86%. There was significant difference in the prevalence of different risk populations according to the predictive values of iPRS prediction model and PRS prediction model (*p* < 0.0001) (Figure 5B). The calibration results showed iPRS prediction model and PRS prediction model both well apply personalized prediction to be used for prevention or clinical decision-making, the Brier score of iPRS prediction model was slightly lower than PRS prediction model (Figure 5C). Considering other calibration indicators, the *p* values for the Spiegelhalter’s z test indicate that iPRS prediction model and PRS prediction model were all well-calibrated (*P* all >0.0500). Results of Cox’s slope and intercept were shown that the slope and intercept for iPRS prediction model were close to 1 and 0, respectively, indicating a proper calibration and a little overestimation of low risk (Cox’s intercept = −1.0771 × 10^−8^), while there was a little underestimation of high risk for PRS prediction model (Cox’s intercept = −8.2160 × 10^−9^). (Supplementary Table S2). The power analyses and computation time of building the iPRS prediction model compared to the traditional PRS prediction model were shown in Supplementary (Supplementary Table S3 and Supplementary Figure S8).

## Discussion

In this manuscript, we proposed a novel PRS construction method and applied it to GWAS analysis scenarios with G×E interactions. Two extensive simulations and 5-fold cross-validation of PLCO real data analysis suggested that iPRS prediction model could improve the prediction performance of disease risk, the accuracy of risk stratification, and clinical calibration performance compared with traditional PRS prediction model. In particular, when the G×E interactions were mainly antagonistic, the predictive accuracy significantly increased. For stratification, according to the predictive value of iPRS prediction model, the prevalence of high-risk populations was significantly higher than that of traditional PRS prediction model. If iPRS prediction model can be applied to disease screening, it can more accurately identify the population with high risk of disease and implement interventions to better prevent or recover. Accuracy prediction, stratification can also reduce costs. Moreover, well-calibration could make the predictive risk obtained according to iPRS prediction model tend to the real risk and promote the development of precision medicine.

The result of our study raised the concern that whether it is sufficient to model the interaction between genetic susceptibilities and environments by using the PRS constructed based solely on the main effects of SNPs. This article was the first to authors’ knowledge to focus on leveraging gene-environment interactions to reconstruct polygenic risk score and to validate the effects of PRS with G×E interactions through simulations and application. When considering gene-environment interactions, most of the current papers used PRS to directly interact with the environment to build the prediction model. (Li et al., 2015; Peyrot et al., 2018; Guloksuz et al., 2019) As we all know, a genetic variation can explain very little heritability, so PRS combines genetic information to estimate a composite score that predicts genetic susceptibility to disease and does not represent any biological significance. Similarly, the interaction between PRS and environment does not have biological significance and cannot represent the interactions between each SNP and environment. IPRS includes interactions between each SNP and environment into the score, respectively. This prevents antagonistic interactions and synergistic interactions from canceling each other out. Furthermore, iPRS uses the main effects of SNPs after considering interaction as the weight of SNPs and the interaction effects of G×E interactions as the weight of G×E interactions, so that we can accurately consider the interactions between each SNP and environment and use their accurate effect as the weight. According to the above, iPRS could improve the prediction performance of PRS prediction model. Finally, 5-fold cross-validation was used to evaluate the performance of the two models in application, which could not only prevent over-fitting, but also proved that the iPRS prediction model was extrapolated.

The effectiveness of risk score is typically assessed by determining whether they can help to stratify populations into different degrees of risk subgroups to drive clinical or individual decisions. (Torkamani et al., 2018) Although several studies have evaluated the risk stratification of PRS, their traditional PRS with missing heritability problem often led to results that were not promising. (Weissfeld et al., 2015; Jia et al., 2020; Tasa et al., 2020) Many explanations for this missing heritability have been suggested, with the two leading causes being rarer variants and gene-environment interactions. (Manolio et al., 2009; Zuk et al., 2012) IPRS incorporates gene-environment interactions into risk score to better explain missing heritability and make prediction effects and risk stratification more precise, demonstrating that GWAS findings could be more accurately used for screening and individualized prevention of complex diseases.

The primary limitation of the proposed method is that the G×E interactions are expected to be as antagonistic as possible. Although the simulation results show that the iPRS prediction model can improve the prediction effect in all three scenarios, the improvement of the prediction effect is not statistically significant when the interaction is synergistic, and it is statistically significant when the interaction is antagonistic. The second limitation of the iPRS is similar to it faced by PRS. The requirement is that individual-level data are available for SNP, environment factor, and outcome. Although summary data can also be used, most GWAS studies only obtained the correlation coefficient between SNPs and outcome, and rarely the correlation coefficient of interaction. The third limitation was that interaction included in iPRS prediction model may be difficult to explain, and to account for genetically determined environmental exposure triggers the disease or not. Since the interaction between SNPs in the same gene set and environment may be opposite, and Many disease-associated SNPs identified to date do not lie in genes (Fridley and Biernacka, 2011), we did not consider interaction between gene set and environment, but interaction between each SNP and environment.

In conclusion, we have proposed a novel PRS construction method applicable to scenarios considering G×E interactions. This proposed method reconstructed PRS by leveraging gene-environment interactions. Compared with the traditional prediction model, iPRS prediction model has better prediction performance, risk stratification, calibration model. Therefore, we expect that iPRS prediction model could have a good application prospect in optimizing the screening criteria of high-risk populations, designating individualized screening programs, and improving the clinical benefits of preventive interventions among populations.

## Data Availability

Publicly available datasets were analyzed in this study. This data can be found here: The data and analyses presented in the current publication are based on the use of study data downloaded from the dbGaP website, under phs000093.v1.p1 and phs000336.v2.p2 (https://www.ncbi.nlm.nih.gov/projects/gap/cgi-bin/study.cgi?study_id=phs000336.v1.p1, https://www.ncbi.nlm.nih.gov/projects/gap/cgi-bin/study.cgi?study_id=phs000093.v2.p2).
