# Supplementary figures and images for: IPRS: Leveraging Gene-Environment Interaction to Reconstruct Polygenic Risk Score
Source: Front Genet. 2022 Mar 24;13:801397. doi: 10.3389/fgene.2022.801397 (PMC8989431; doi:10.3389/fgene.2022.801397)

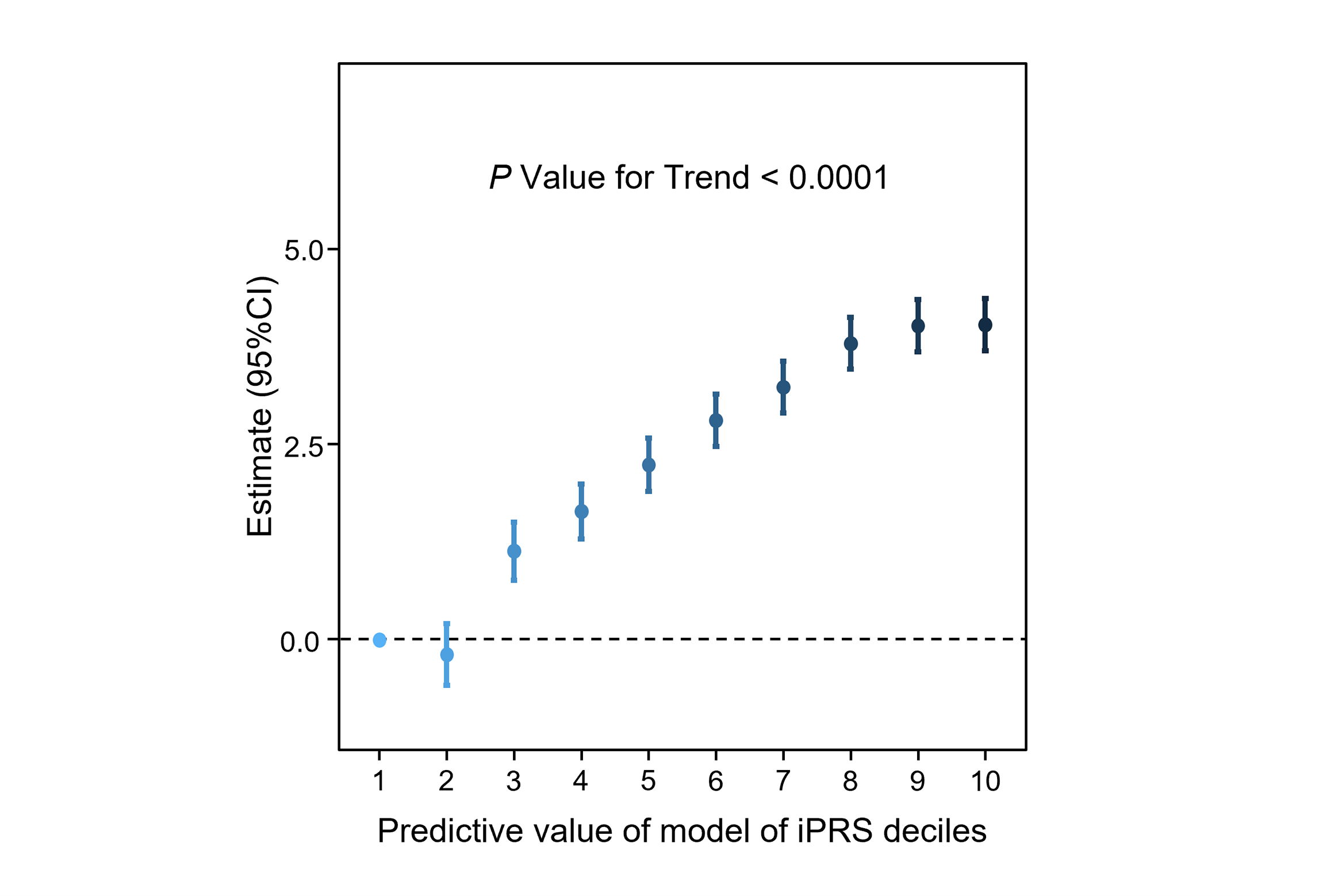

Supplement: Supplementary file 1 [file Image6.TIF]

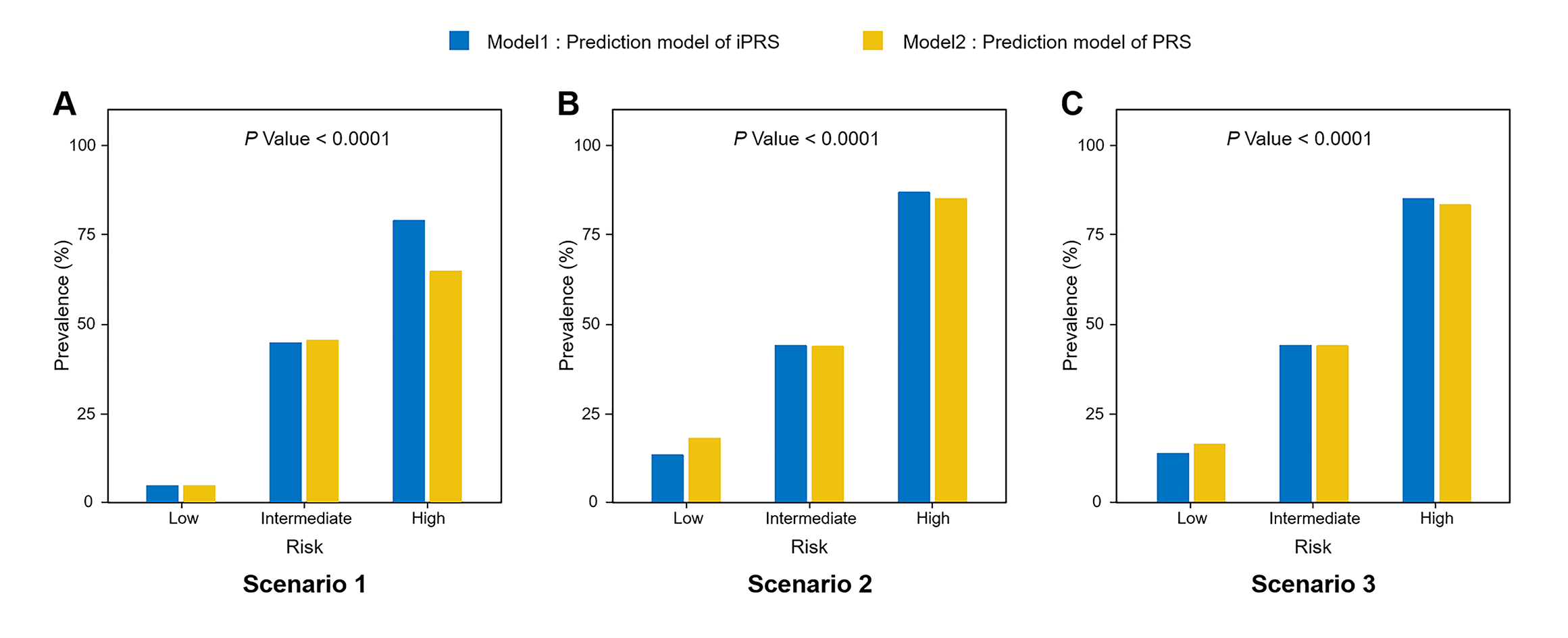

Supplement: Supplementary file 3 [file Image3.TIF]

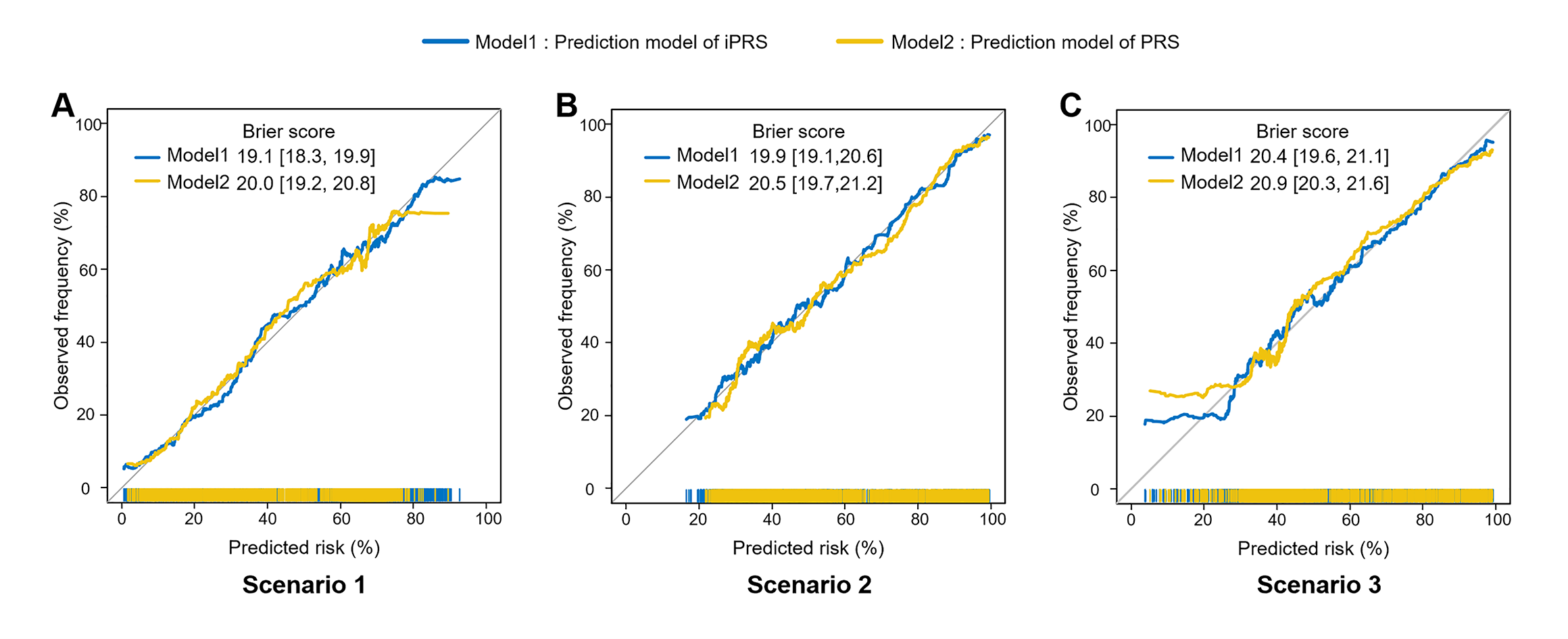

Supplement: Supplementary file 4 [file Image4.TIF]

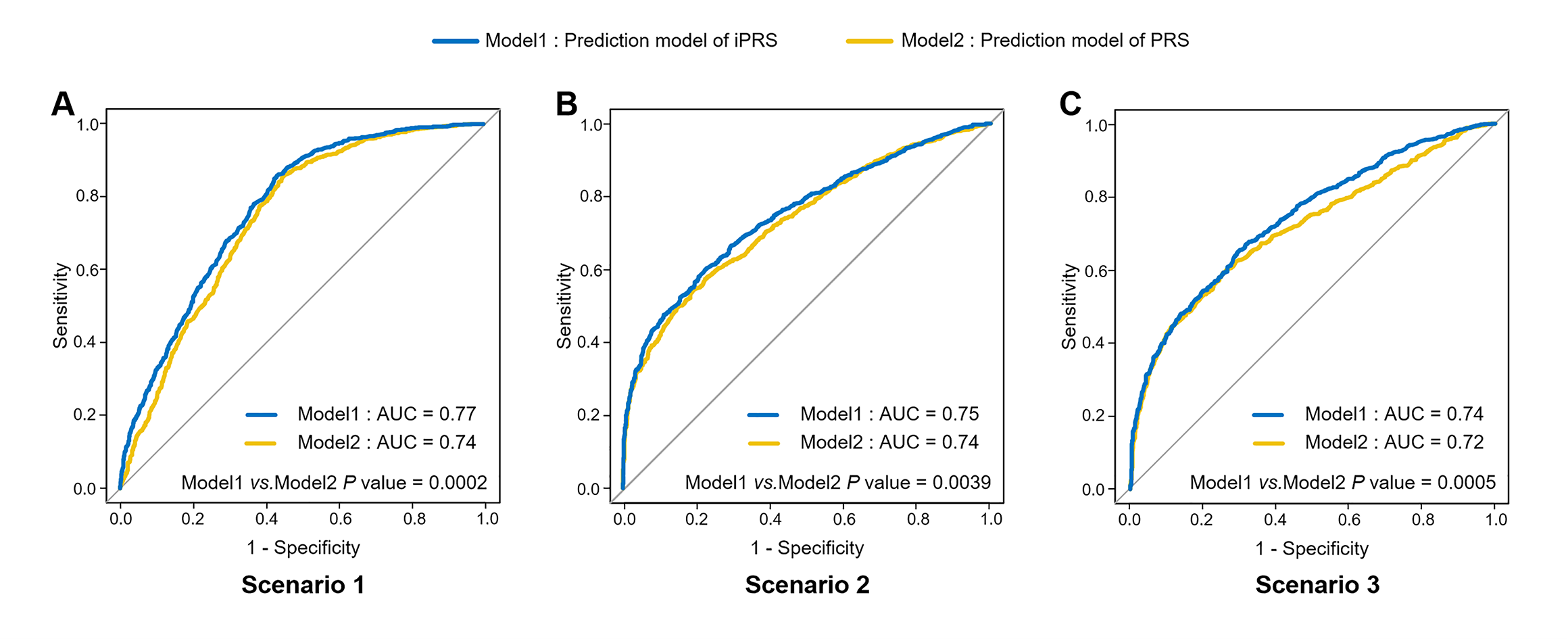

Supplement: Supplementary file 5 [file Image2.TIF]

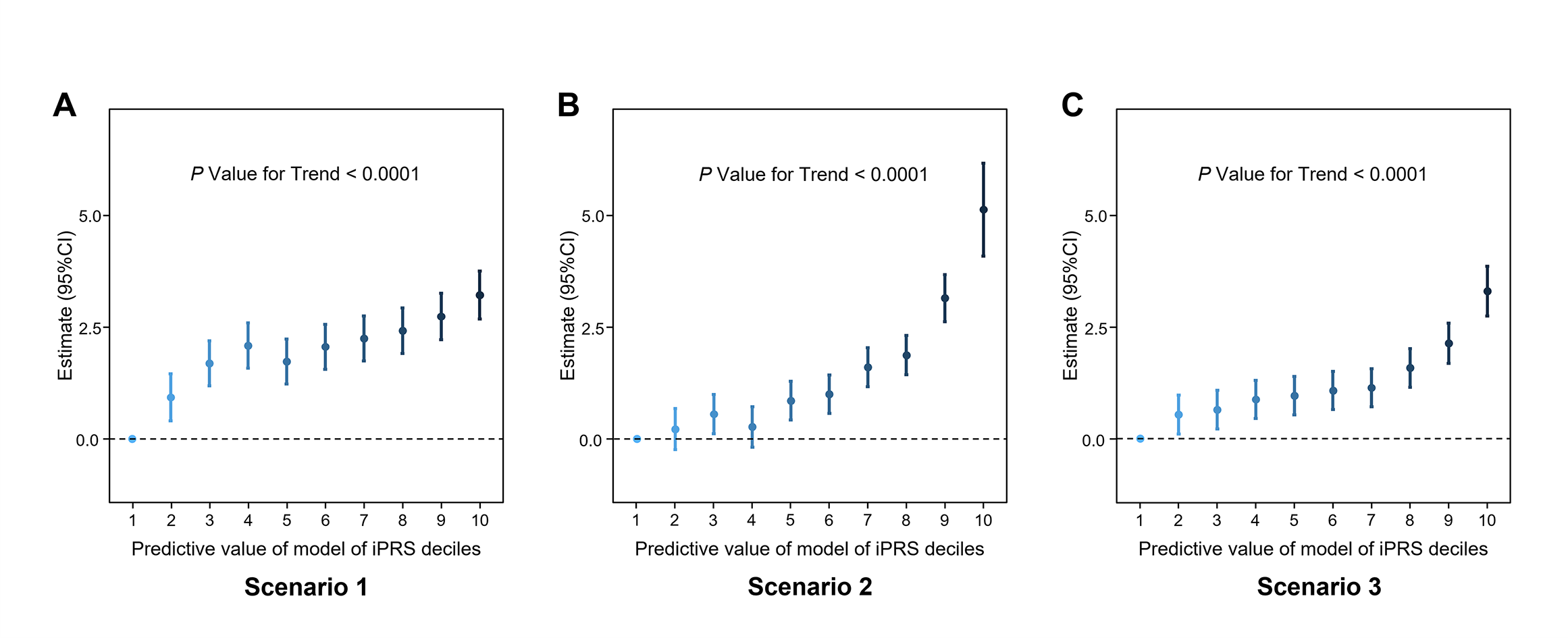

Supplement: Supplementary file 6 [file Image1.TIF]

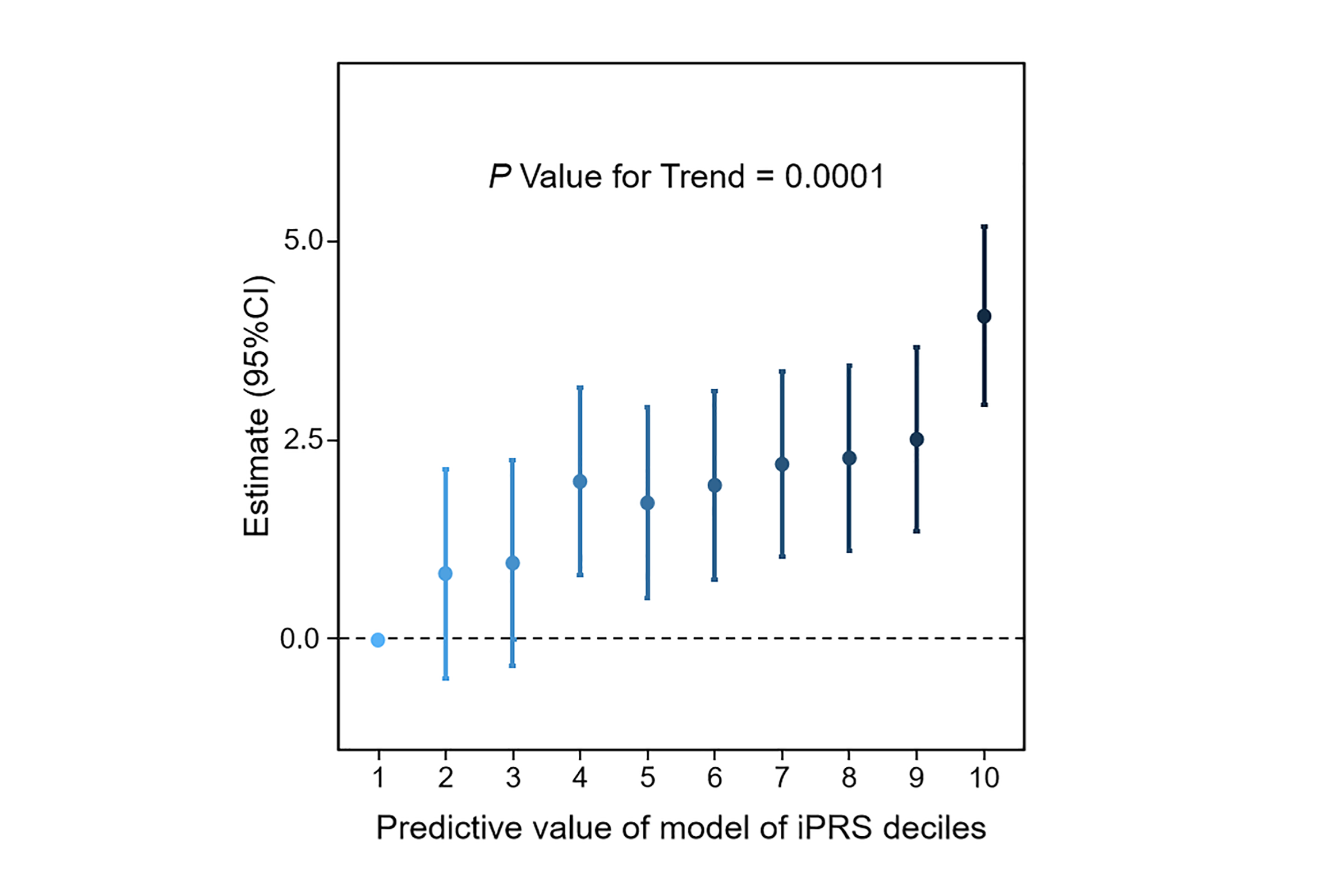

Supplement: Supplementary file 7 [file Image7.TIF]

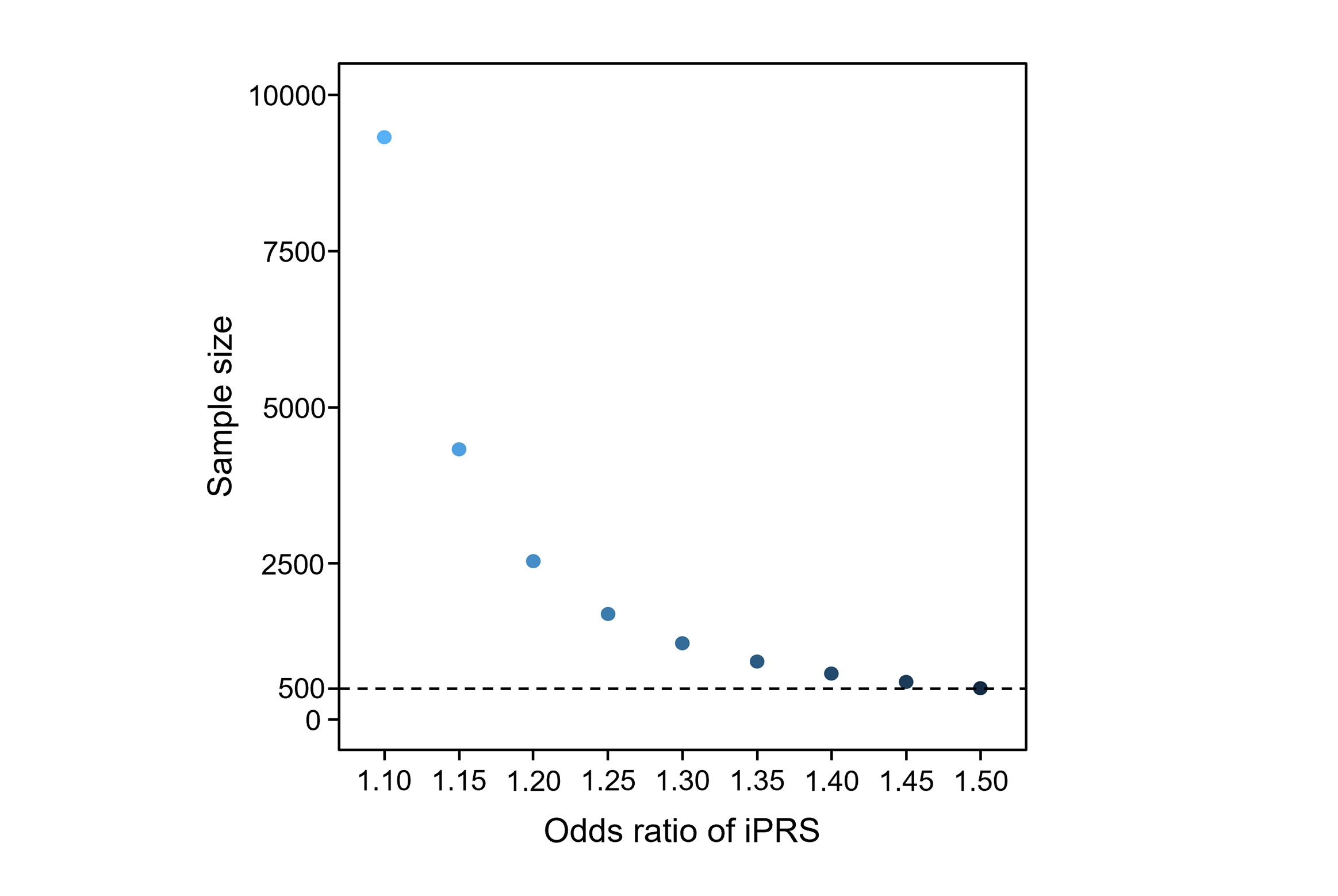

Supplement: Supplementary file 8 [file Image8.TIF]

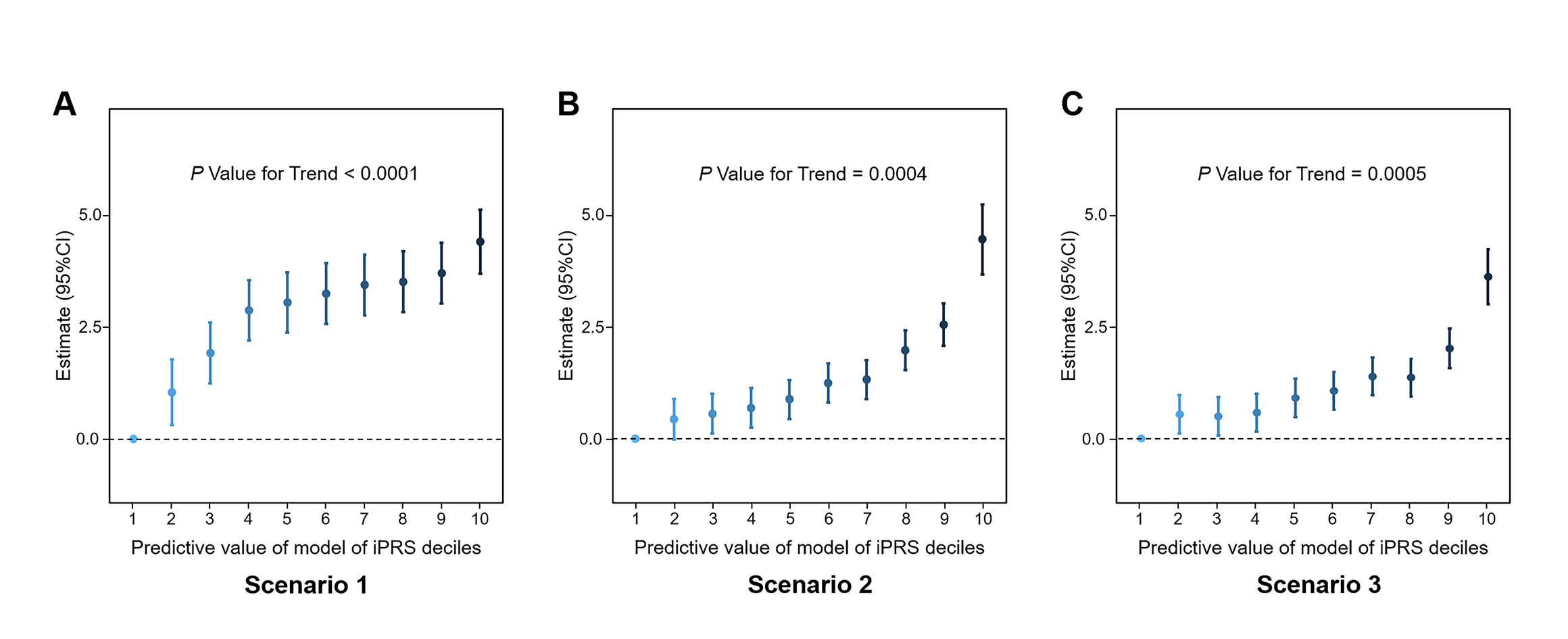

Supplement: Supplementary file 9 [file Image5.TIF]
