# Supplementary material for: IPRS: Leveraging Gene-Environment Interaction to Reconstruct Polygenic Risk Score
Source: Front Genet. 2022 Mar 24;13:801397. doi: 10.3389/fgene.2022.801397 (PMC8989431; doi:10.3389/fgene.2022.801397)
Supplement: Supplementary file 2 [file Table1.docx]

Supplementary Material

# Supplementary Figures and Tables

## Supplementary Figures


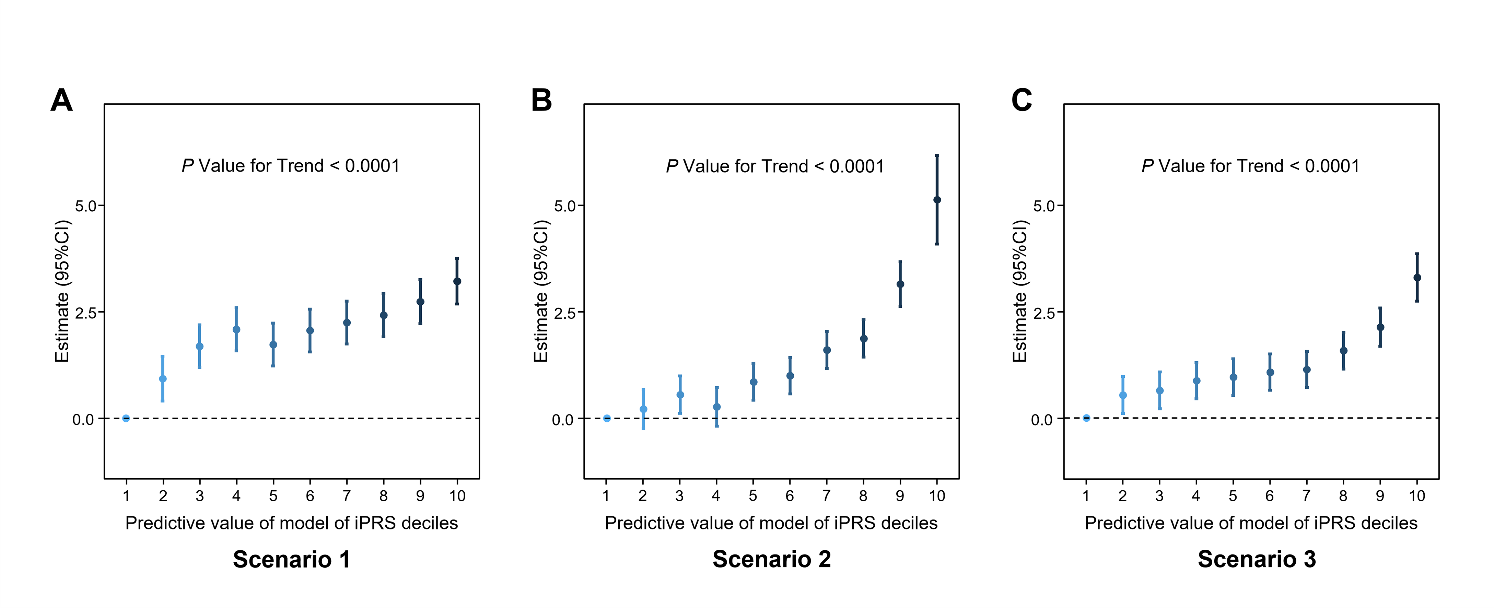


**Supplementary Figure S1 Risk for disease according to iPRS in 3 scenarios of simulation I with dependent sparse SNPs.** IPRS and the risk of disease shape plot representing the risk of iPRS on the estimated risk of disease in simulation I (1,000 cases, 1,000 controls). The iPRS has been divided into 10 deciles based on iPRS values. The reference group is the lowest decile (0–10%). Estimated disease risks are shown as blue dots, with their respective 95th percent confidence intervals displayed as blue vertical lines. The color deepens as the iPRS decile increases. The dashed line is the reference at the bottom iPRS decile (estimated disease risk = 0).

**
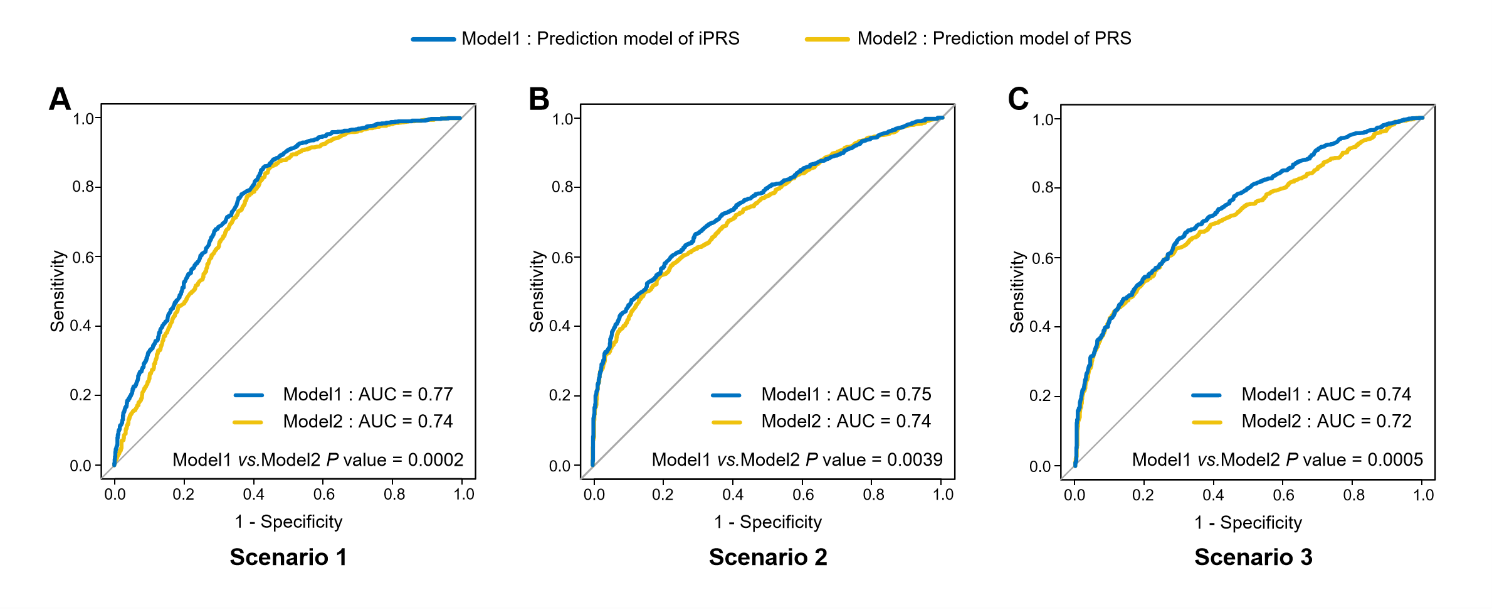
**

**Supplementary Figure S2 ROC of iPRS prediction model and PRS prediction model in 3 scenarios of additional simulations. (A)** Scenario 1: SNPs are all risk factors, the environmental variant is a risk factor, interactions are antagonistic; **(B)** Scenario 2: SNPs are all risk factors, the environmental variant is a risk factor, interactions are synergistic; **(C)** Scenario 3: SNPs are all risk factors, the environmental factor is a risk variant, half of the interactions are antagonistic, and half are synergistic. The *P* values were shown that the results of comparing the AUC of iPRS prediction model and traditional PRS prediction model.


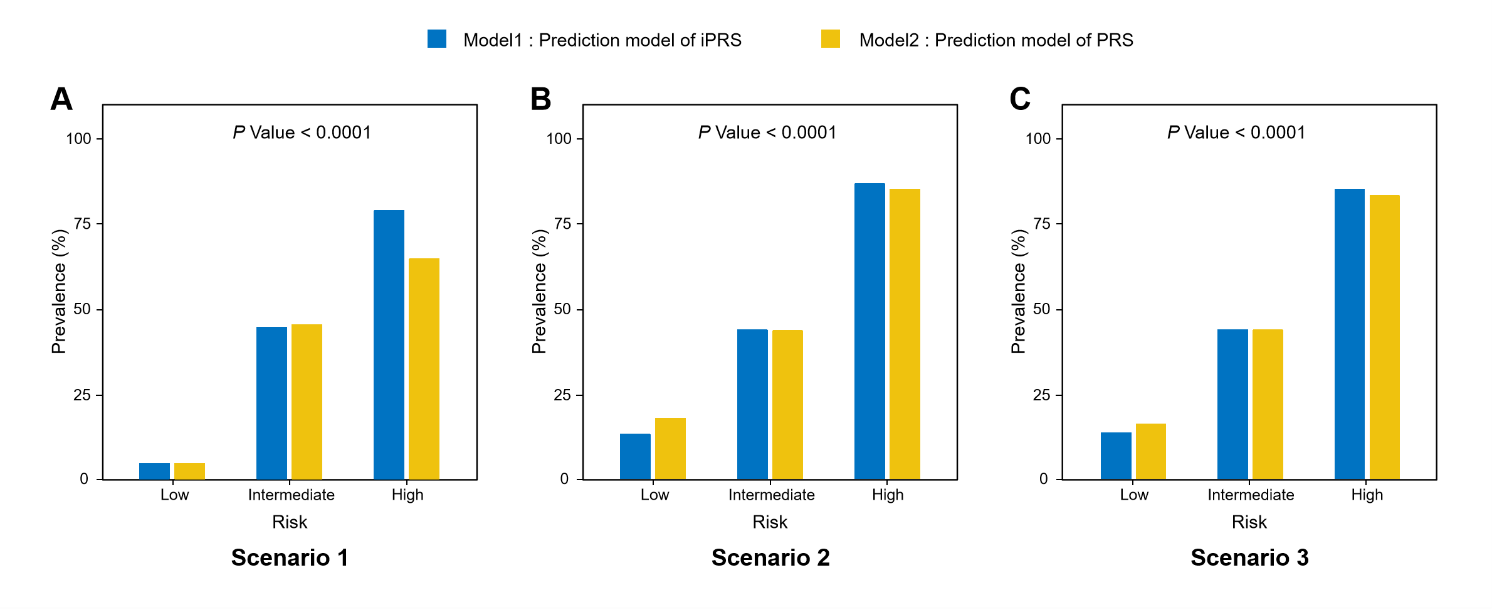


**Supplementary Figure S3** **Prevalence of high risk, intermediate risk, low risk population according to the predictive value of iPRS prediction model or PRS prediction model in 3 scenarios of additional simulations.** Samples were defined as high risk, intermediate risk, and low risk populations according to the top 5%, 5%-95%, and the bottom 5% of the predictive value of iPRS prediction model or PRS prediction model. Significant differences in risk categories of the population were noted. **(A)** Scenario 1: SNPs are all risk factors, the environmental variant is a risk factor, interactions are antagonistic; **(B)** Scenario 2: SNPs are all risk factors, the environmental variant is a risk factor, interactions are synergistic; **(C)** Scenario 3: SNPs are all risk factors, the environmental factor is a risk variant, half of the interactions are antagonistic, and half are synergistic.


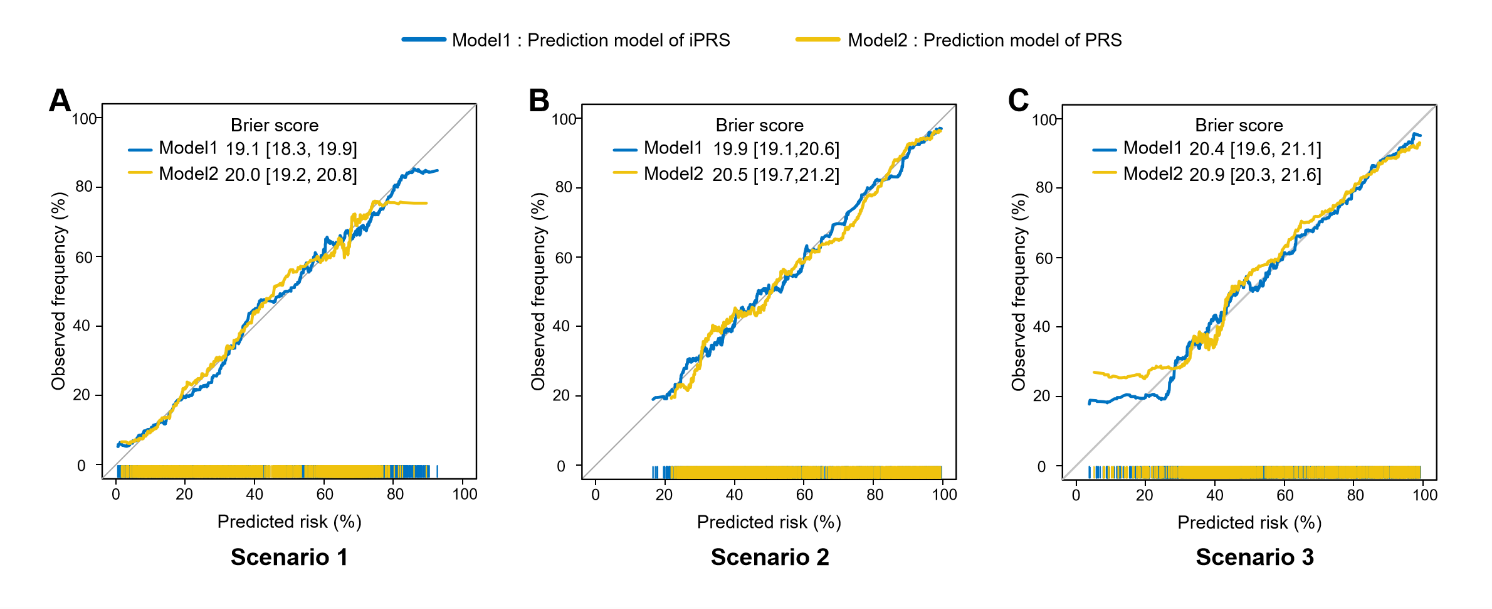


**Supplementary Figure S4 Calibration Plots and Brier scores of iPRS prediction model and PRS prediction model in 3 scenarios of additional simulations.** Calibration plots summarize the graphical agreement between observed and predicted risks. In an ideal model, pairs of the observed and predicted risks lie on a 45-degree angle line. Curves falling under the 45-degree angle line indicate that predicted risks overestimate (are higher than) observed risks, while curves falling above the 45-degree angle line indicate that predicted risks underestimate (are lower than) observed risks. **(A)** Scenario 1: SNPs are all risk factors, the environmental variant is a risk factor, interactions are antagonistic; **(B)** Scenario 2: SNPs are all risk factors, the environmental variant is a risk factor, interactions are synergistic; **(C)** Scenario 3: SNPs are all risk factors, the environmental factor is a risk variant, half of the interactions are antagonistic, and half are synergistic. The numbers in square brackets represent 95%CI of the Brier score.


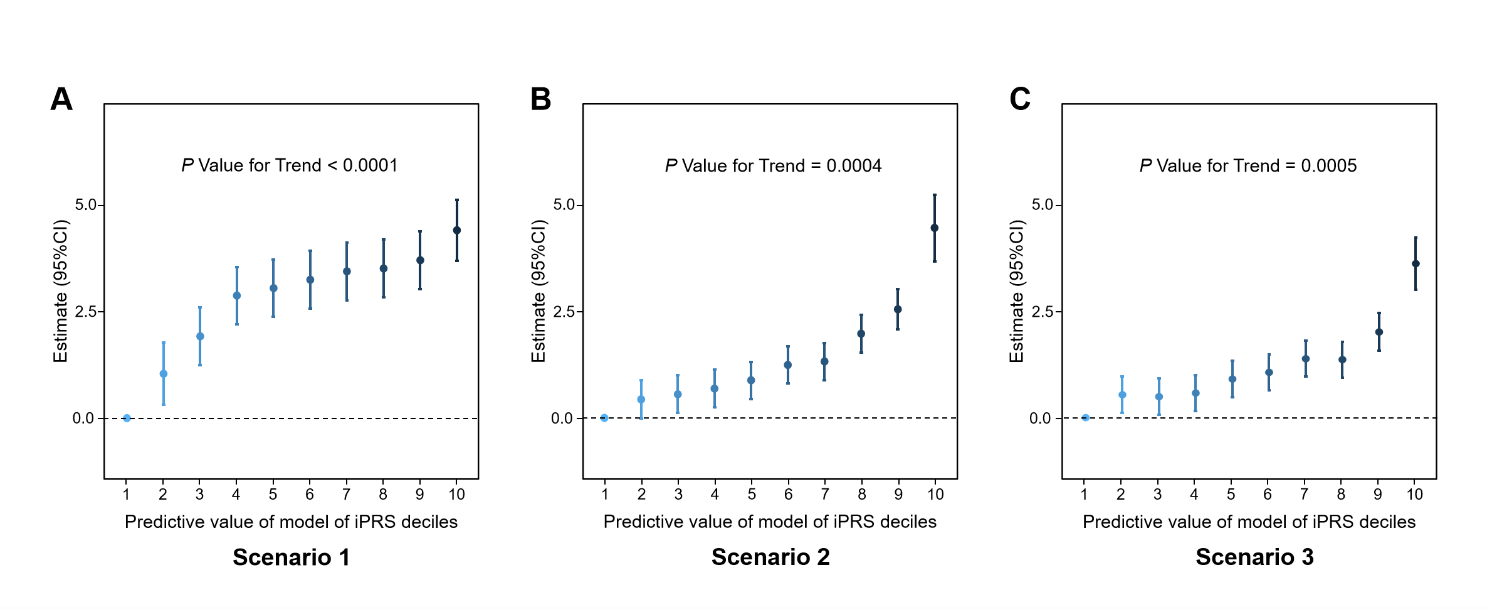


**Supplementary Figure S5 Risk for disease according to iPRS in 3 scenarios of additional simulations.** IPRS and the risk of disease shape plot representing the risk of iPRS on the estimated risk of disease in simulation I (1,000 cases, 1,000 controls). The iPRS has been divided into 10 deciles based on iPRS values. The reference group is the lowest decile (0–10%). Estimated disease risks are shown as blue dots, with their respective 95th percent confidence intervals displayed as blue vertical lines. The color deepens as the iPRS decile increases. The dashed line is the reference at the bottom iPRS decile (estimated disease risk = 0).


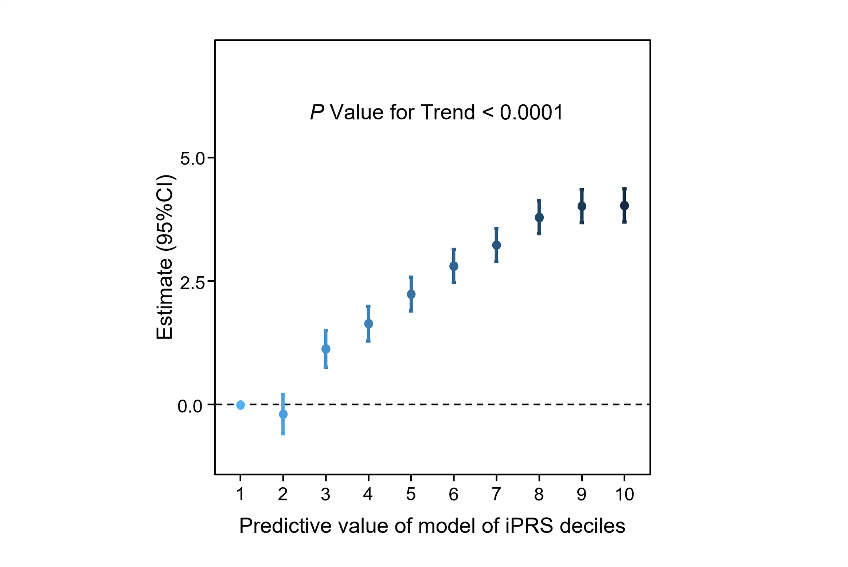


**Supplementary Figure S6 Risk for disease according to iPRS in 3 scenarios of simulation II with real LD structure genotypes.** Estimated disease risks of iPRS in simulation II (with 5,333 cases and 9,082 controls) for each iPRS decile. The square dots are the estimated disease risks of iPRS, and the error bars are the 95% CI. The dashed line is the reference at the bottom iPRS decile (estimated disease risks = 0).


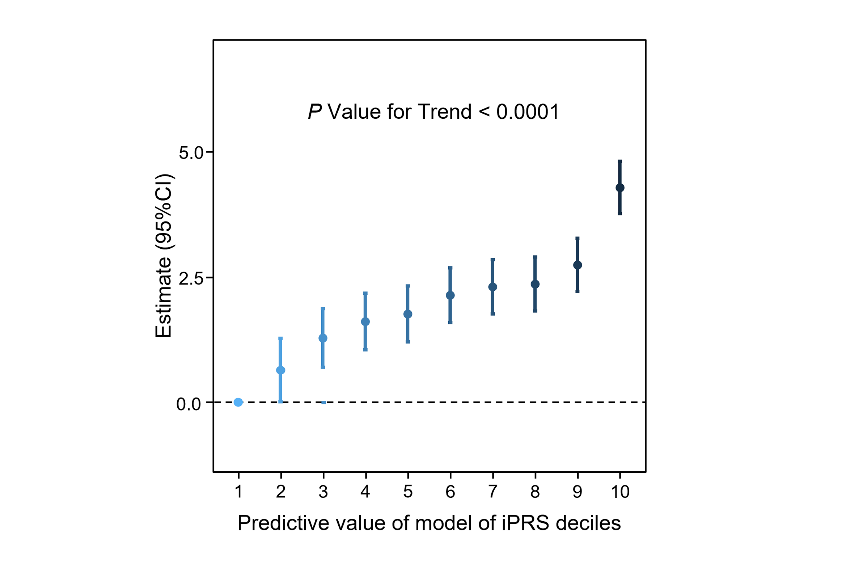


**Supplementary Figure S7 Risk for disease according to iPRS in the application of PLCO study.** Estimated disease risks of iPRS in the application of PLCO (with 1,453 lung cancer cases and 12,692 controls) for each iPRS decile. The square dots are the estimated disease risks of iPRS, and the error bars are the 95% CI. The dashed line is the reference at the bottom iPRS decile (estimated disease risks = 0).


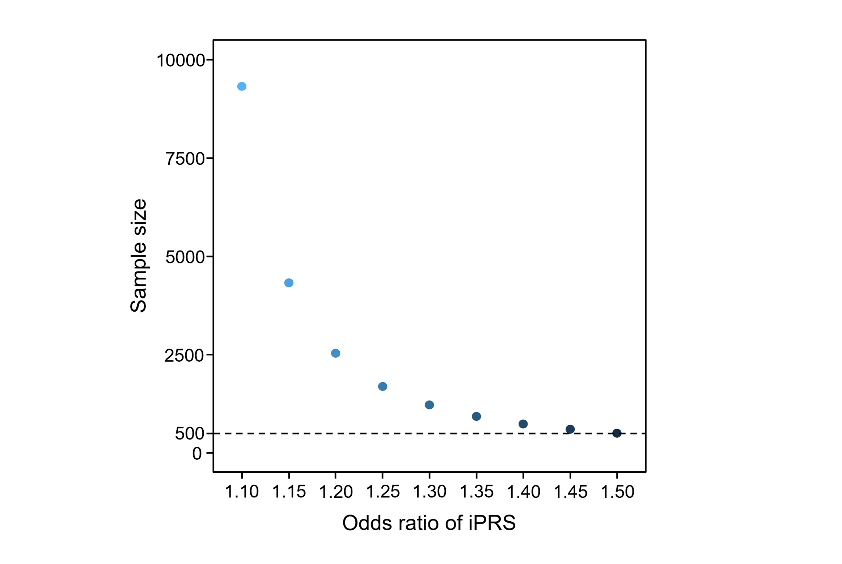


**Supplementary Figure S8 Relationship between Sample size and Odds ratio of iPRS in iPRS prediction model under the substantial power (say 80%).** Sample sizes were calculated corresponding to different odds ratios of iPRS in iPRS prediction model under the condition of substantial power(say 80%). The required sample size decreased with the increase of odds ratio of iPRS, especially the sample size was less than 500 (dashed line) when odds ratio of iPRS was greater than 1.50. The statistical power and sample size analyses were performed by G*Power software.

## Supplementary Tables

**Supplementary Table S1 Description of simulation I scenarios used in evaluations of the iPRS prediction method and comparisons to existing methods.**

| **Scenario** | **Description** | **Parameter Settings** |
| --- | --- | --- |
| 1 | SNPs are all risk factors, environmental variant is a risk factor, interactions are antagonistic | $\beta_{G_{1}}^{M}$=ln(1.5), $\beta_{G_{2}}^{M}$=ln(1.3), $\beta_{G_{3}}^{M}$=ln(1.7),  $\beta_{G_{4}}^{M}$=ln(1.4), $\beta_{G_{5}}^{M}$=ln(1.3), $\beta_{G_{6}}^{M}$=ln(1.5),  $\beta_{G_{7}}^{M}$=0, $\beta_{G_{8}}^{M}$=0, $\beta_{G_{9}}^{M}$=0, $\beta_{G_{10}}^{M}$=0;  $\beta_{E}$=ln(2.5);$\beta_{G_{1}\times E}^{I}$=ln(1.5), $\beta_{G_{2}\times E}^{I}$=ln(1.3), $\beta_{G_{3}\times E}^{I}$=ln(1.7), $\beta_{G_{4}\times E}^{I}$=ln(1.4), $\beta_{G_{5}\times E}^{I}$=0, $\beta_{G_{6}\times E}^{I}$=0, $\beta_{G_{7}\times E}^{I}$=0, $\beta_{G_{8}\times E}^{I}$=0, $\beta_{G_{9}\times E}^{I}$=0, $\beta_{G_{10}\times E}^{I}$=0. |
| 2 | SNPs are all risk factors, environmental variant is a risk factor, interactions are synergistic | $\beta_{G_{1}\times E}^{I}$=ln(2), $\beta_{G_{2}\times E}^{I}$=ln(4), $\beta_{G_{3}\times E}^{I}$=ln(2.5), $\beta_{G_{4}\times E}^{I}$=ln(3), $\beta_{G_{5}\times E}^{I}$=0, $\beta_{G_{6}\times E}^{I}$=0, $\beta_{G_{7}\times E}^{I}$=0, $\beta_{G_{8}\times E}^{I}$=0, $\beta_{G_{9}\times E}^{I}$=0, $\beta_{G_{10}\times E}^{I}$=0;  the other parameters are set as above. |
| 3 | SNPs are all risk factors, environmental factor is a risk variant, half of interactions are antagonistic, and half are synergistic | $\beta_{G_{1}\times E}^{I}$=ln(1.5), $\beta_{G_{2}\times E}^{I}$=ln(1.3), $\beta_{G_{3}\times E}^{I}$=ln(1.7), $\beta_{G_{4}\times E}^{I}$=ln(1.4), $\beta_{G_{5}\times E}^{I}$=0, $\beta_{G_{6}\times E}^{I}$=0, $\beta_{G_{7}\times E}^{I}$=0, $\beta_{G_{8}\times E}^{I}$=0, $\beta_{G_{9}\times E}^{I}$=0, $\beta_{G_{10}\times E}^{I}$=0;  the other parameters are set as above. |

**Supplementary Table S2 Discrimination and calibration results of the iPRS prediction model and PRS prediction model applied to PLCO.**

| **Calibration Indexes** | **iPRS prediction model** | **PRS prediction model** |
| --- | --- | --- |
| Brier score | 0.0622 | 0.0619 |
| Spiegelhalter z score | -1.3651 | 0.0793 |
| Spiegelhalter *P* value | 0.0861 | 0.4684 |
| Average absolute error | 0.1265 | 0.1236 |
| Cox’s slope | 1.0000 | 1.0000 |
| Cox’s intercept | -1.0771×10^-8^ | 8.2160×10^-9^ |
| MCE | 0.0232 | 0.0209 |
| ECE | 0.0067 | 0.0073 |
| ICI | 0.0083 | 0.0080 |

The Brier score is a combined measure of discrimination and calibration. Calibration also is measured by the Spiegelhalter *z* test, average absolute error, MCE, ECE, Cox slope and intercept, and ICI.

IPRS: interaction polygenic risk scores; PRS: polygenic risk scores; ECE: expected calibration error; MCE: maximum calibration error; ICI: integrated calibration index.

**Supplementary Table S3 Statistical power analyses and computation time of the iPRS prediction model and PRS prediction model applied to PLCO for 5-fold cross-validation.**

|  | **iPRS prediction model** | **PRS prediction model** |
| --- | --- | --- |
| Statistical power (%) | 97.01 | 86.00 |
| Computation time (mins) | 56.43 | 42.93 |

The statistical power analyses were performed by G*Power software.

The computation times were calculated by R software.
